# High Abdominal Perfusion Pressure Using Umbilical Cord Flap in the Management of Gastroschisis

**DOI:** 10.3389/fped.2021.706213

**Published:** 2021-09-30

**Authors:** Mohamed Ahmed Arafa, Khalid Mohamed Elshimy, Mohamed Ali Shehata, Akram Elbatarny, Hisham Almohamady Almetaher, Hamed Mahmoud Seleim

**Affiliations:** Pediatric Surgery Unit, Faculty of Medicine, Tanta University, Tanta, Egypt

**Keywords:** gastroschisis, perfusion, inspiratory, venous pressure, tension-free

## Abstract

**Background:** Gastroschisis management remains a controversy. Most surgeons prefer reduction and fascial closure. Others advise staged reduction to avoid a sudden rise in intra-abdominal pressure (IAP). This study aims to evaluate the feasibility of using the umbilical cord as a flap (without skin on the top) for tension-free repair of gastroschisis.

**Methods:** In a prospective study of neonates with gastroschisis repaired between January 2018 to October 2020 in Tanta University Hospital, we used the umbilical cord as a flap after the evacuation of all its blood vessels and suturing the edges of the cord with the skin edges of the defect. They were guided by monitoring abdominal perfusion pressure (APP), peak inspiratory pressure (PIP), central venous pressure (CVP), and urine output during 24 and 48 h postoperatively. The umbilical cord flap is used for tension-free closure of gastroschisis if PIP > 24 mmHg, IAP > 20 cmH_2_O (15 mmHg), APP <50 mmHg, and CVP > 15cmH_2_O.

**Results:** In 20 cases that had gastroschisis with a median age of 24 h, we applied the umbilical cord flap in all cases and then purse string (Prolene Zero) with daily tightening till complete closure in seven cases, secondary suturing after 10 days in four cases, and leaving skin creeping until complete closure in nine cases. During the trials of closure, the range of APP was 49–52 mmHg. The range of IAP (IVP) was 15–20 cmH_2_O (11–15 mmHg), the range of PIP was 22–25 cmH_2_O, the range of CVP was 13–15 cmH_2_O, and the range of urine output was 1–1.5 ml/kg/h.

**Conclusion:** The umbilical cord flap is an easy, feasible, and cheap method for tension-free closure of gastroschisis with limiting the PIP ≤ 24 mmHg, IAP ≤ 20 cmH_2_O (15 mmHg), APP > 50 mmHg, and CVP ≤ 15cmH_2_O.

## Introduction

Gastroschisis is a developmental abdominal wall defect in which the bowel and /or other organs herniate without coverings or sac (1). The incidence of gastroschisis has ascended from 1/4,000 to 1/1,000 live births with a noticeable correlation with young mothers (2).

It was mentioned over many years that the primary closure was the correction of choice because staged closure had an increased risk of infection and delayed return to normal intestinal function in addition to prolonged hospital stay (3).

Bearing on the viscero-abdominal discrepancy noticed in a considered number of cases, in addition, the orientation of abdominal compartment syndrome (ACS) together with the improvement of surgical methods, the rationale of delayed primary or staged closure has been accepted in patients with specific criteria (4–6).

Normal intra-abdominal pressure (IAP) in neonatal age is about 10 mmHg, whereas intra-abdominal hypertension (IAH) is defined as a sustained rise of IAP > 10 mmHg (3). ACS is a sustained IAP of >10 mmHg associated with organ dysfunction (7, 8). Abdominal perfusion pressure (APP) is more accurate in the evaluation of visceral blood supply and a need for resuscitation. Its normal range is from 40 to 50 mmHg (9).

Initially, the treatment of neonates with gastroschisis was rapid reduction of the viscera and primary fascial closure. Although this can be accomplished in some cases, primary closure is often not possible as the size of the defect is too large, and it may result in compromise of venous return and respiration. Also, delayed primary closure after using a silo may be complicated by dehiscence and infection, which requires removal of the prosthesis. When one or more of these complicating issues are present, early fascial closure is impossible, and the treatment priority is the protection of the exposed viscera. This may be done by covering the viscera with flaps. The large ventral hernia created in this way may require delayed herniorrhaphy (10).

Intravesical pressure (IVP), splanchnic perfusion pressure, and ventilatory indices have been developed as different parameters upon which primary or staged closure should be taken; however, the selection of operative procedure of gastroschisis is still controversial (11–13).

In 1970, the umbilical cord as autogenic material was considered useful for the repair of experimentally induced abdominal wall defects in rats (11). Moreover, a full-thickness umbilical cord was successfully used to close the defect in a case with gastroschisis and then the skin was used for coverage on top of it in 1974 (14–17). This study aims to evaluate the feasibility of using the umbilical cord as a flap (without skin on the top) for tension-free repair of gastroschisis.

## Patients and Methods

This study is a prospective review of 20 neonates presented with gastroschisis to Tanta University Hospital from January 2018 to October 2020. We included all neonates suffering from gastroschisis repaired using the umbilical cord as a flap for tension-free repair. We excluded all cases in which primary fascial closure was done. This was approved by the research ethics committee, Faculty of Medicine, Tanta University, Egypt—Approval code: 311467/7/9. All patients included in this study gave written informed consent to participate in this research by their parents or legal guardian. Upon our operative plane, we instructed obstetricians to preserve all lengths of the umbilical cord by cutting it at the placenta and covering it with the bowel by warm dressing. A nasogastric tube was used to decompress the stomach in addition to warming of the neonate and then transfer to the operating room (OR) after stabilization. A central venous line was inserted in the internal jugular or subclavian vein. Under general anesthesia, the bowel is inspected for ischemia, perforation, atresia, and other malformations. The intestine was then decompressed by evacuating the meconium to decrease the distension. Reduction of the gut into the abdomen and primary fascial closure was tried by putting stay sutures on both edges of the sheath and trying of approximation while monitoring IAP through IVP measurement, APP, peak inspiratory pressure (PIP), central venous pressure (CVP), and urine output.

The umbilical cord flap is used for tension-free closure of gastroschisis if PIP > 24 mmHg, IAP > 20 cmH_2_O (15 mmHg), APP <50 mmHg, and CVP > 15 cmH_2_O.

1. Measurement of IVP as follows: Initially, before the procedure, a six French feeding tube was inserted for complete emptying of the bladder. The catheter was connected by a three-way stopcock to a vertical plastic tube. The system was filled with saline and allowed to equilibrate. The pressure was considered zero when the saline was at the level of the symphysis pubis in the mid-axillary line at the level of the iliac crest, and then the height of the column of saline was measured at the end of expiration. All pressures were noted in centimeters of saline. If IVP remained under 20 cmH_2_O (15 mmHg), the sutures were tied, and the fascia was closed as usual. If IVP was > 20 cmH_2_O, the umbilical cord flap was used.
2. Measurement of APP: A visceral perfusion indicator indicated good resuscitation. It is considered normal from 40 to 50 mmHg.
  - **APP** **=** **MAP – IAP**
  - Mean arterial pressure (MAP) = (systolic blood pressure + 2 × diastolic blood pressure)/3.
  - It must be ≥50 mmHg to maintain adequate tissue perfusion.
  - If APP > 50 mmHg, the fascial closure was done as usual. If APP is <50 mmHg, an umbilical cord flap was used.
3. Measurement of PIP is the total amount of airway pressure delivered by the ventilator to overcome resistive work by the lung to open its alveoli. If PIP remained under 24 cmH_2_O, the sutures were tied, and closure of the fascia was done as usual. If PIP was higher than 24 cmH_2_O, an umbilical cord flap was used.
4. Measurement of CVP after insertion of a central line: If CVP remained <15 cmH_2_O, the fascia was closed as usual. If PIP was > 15 cmH_2_O, an umbilical cord flap was used.

### Operative Steps

**Figure 1:**
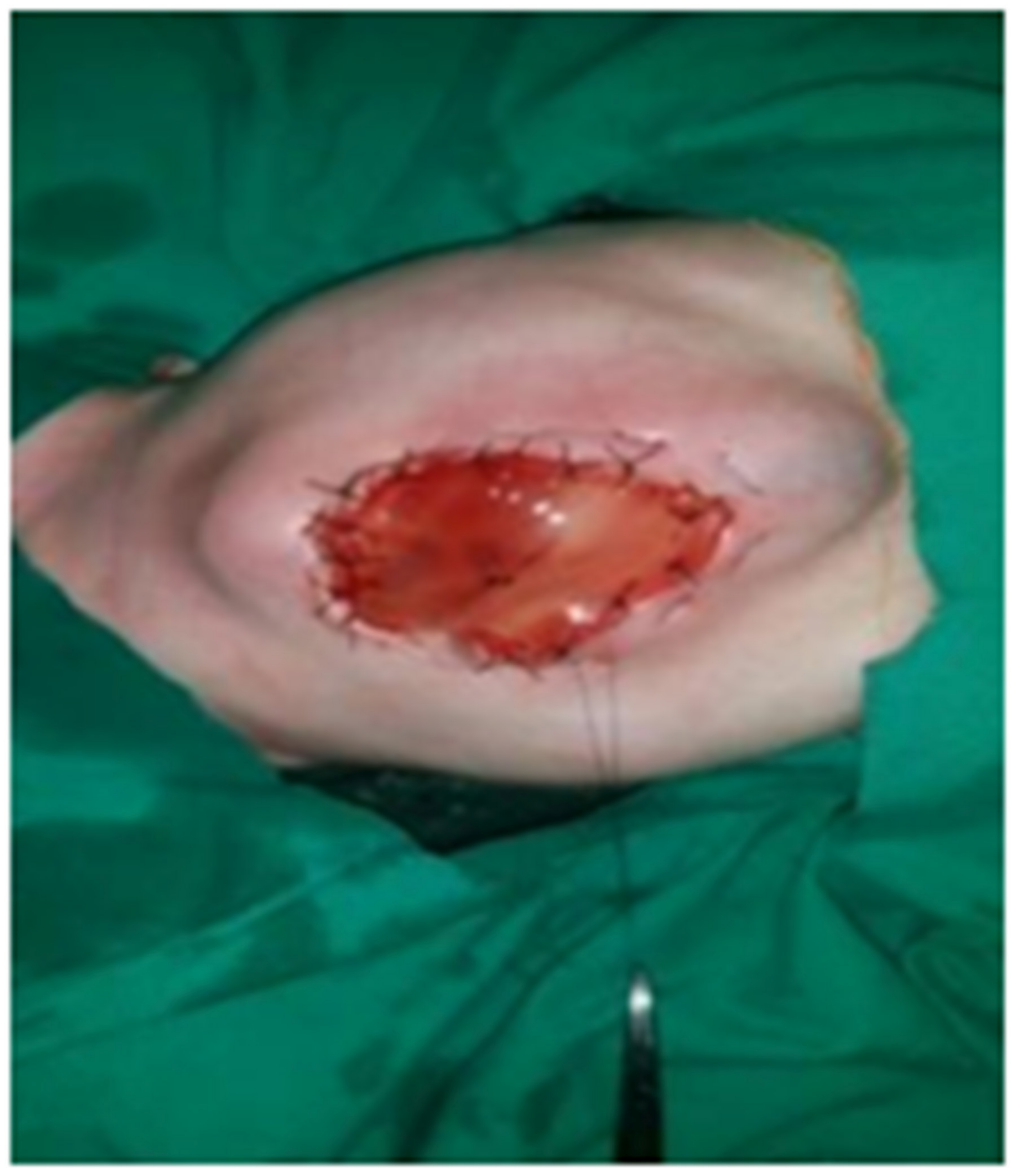
Use of umbilical cord flap with purse string.

We split the umbilical cord vertically to lay it open and then removed all contained vessels. The edges of the cord are sutured around the defect with the fascia and skin using interrupted stitches. In cases with large defects, it may be necessary to fold the umbilical cord flap back and suture the edges of the flap to themselves in the middle of the defect while suturing the periphery of the flap to the facia and skin. In some cases with small defects, we apply purse strings (Prolene Zero) on the edges of the skin with daily tightening till complete closure (Figure 1).

A moist bandage soaked with topical antibiotics was used to cover the umbilical cord flap, and then the neonate was transferred to the NICU. IVP, APP, PIP, CVP, and urine output are monitored in the following postoperative 2 days. Extubation is possible after 2 days and when breathing is adequate. The flap is swabbed every 6 h with an antiseptic solution and covered with a moist dressing. Parenteral antibiotics were also administered according to the severity of the case. A few days postoperatively, the umbilical cord shrinks due to water loss. Together with gravity, this leads to a continuous reduction of the bowel. We start enteral feeding once we observe intestinal movement.

Statistical methods: Data was recorded and analyzed using Microsoft Excel 2019® spreadsheets. For statistical analysis, relevant data was transferred to IBM SPSS 20®. Nominal and ordinal data are displayed as numbers and percentages. Metric data are shown as mean and standard deviation. Using SPSS, descriptive analysis showed normal distribution on the blot graph, and the evaluation was the mean as well as standard deviation. Logistic regression analysis and 95.0% CI for odds were used to test the correlation of multiple factors in relation to each other.

## Results

Of 32 cases with gastroschisis during the period of research, in 20 cases, we used an umbilical cord flap in the coverage of a defect when primary closure was not appropriate. The mean age at the surgery, mean gestational age, gender distribution, birth weight, the size of the defect, length of the umbilical cord, duration of hospital stay, and length of parenteral nutrition are tabulated in Table 1. Prenatal diagnosis, mode of delivery, period of ventilation (days), associated defects, and beginning of oral feeding are tabulated in Table 2.

**Table 1:** Demographic, anatomical, and hospital data of our cases.

**Variables**		**Mean ± SD Range %**
Age at time of surgery (days)		1.1 ± 1.8
Gestational age (weeks)		34.8 ± 1.5 (28–37)
Sex	Male	11 (55%)
	Female	9 (45%)
Birth weight (kg)		2.0 ± 0.3 (1.4–2.6)
The size of the defect (cm^2^)		3.2 ± 1.2 (2.6–4.8)
Cord length (cm)		13.4 ± 3.1 (10–22)
Length hospital stay (days)		14.5 ± 2.4 (12–25)
Length of parenteral nutrition (days)		10.5 ± 2.4 (8–17)

**Table 2:** Pre and postnatal history.

**Variables**		**Mean ± SD Range %**
Prenatal diagnosis		8 (40%)
Delivery	Cesarean	14 (70%)
	Vaginal	6 (30%)
Period of ventilation (days)	Preoperative	1.0 ± 0.2 (1–1.5)
	Postoperative	5.5 ± 3.4 (2–12)
Associated	Cardiovascular	9 (45%)
defects	Intestinal atresia	1 (5%)
Beginning of oral feeding		6.5 ± 2.6 (3–11)

Table 3 shows the mean values and ranges of the variable readings of the APP, IVP, PIP, CVP, and urine output per hour during attempts of closure and 24 and 48 h postoperative. The mean IAP shows lower values of pressure in evaluation postoperatively than during operative attempts of closure, but the mean APP shows higher values during the postoperative period than during attempts of operative closure. The mean measurements of PIP and CVP in all postoperative measurements show lower values than during attempts of closure, and urine output improved postoperatively in the following days. All these measurements show improvement in intestinal perfusion and a decrease in intra-abdominal pressure following closure using umbilical cord flap.

**Table 3:** Ranges and the mean values of the different readings of the APP, IVP, PIP, CVP, and urine output per hour during attempts of closure and 24, 48 h postoperative.

**Variables**	**Immediate after complete closure**	**24 h post-operative**	**48 h post-operative**	***P***
APP	50.7 ± 2.4	52 ± 3.1	57 ± 1.8	0.006^*^
	(49–52) mmHg	(51–56) mmHg	(52–59) mmHg	
IVP	17.6 ± 2.4	16.6 ± 2.2	15.2 ± 1.8	0.013^*^
	(15–20) cmH_2_O	(14–19) cmH_2_O	(13–18) cmH_2_O	
PIP	23.8 ± 1.3	21 ± 1.2	19.6 ± 1.4	0.005^*^
	(22–25) cmH_2_O	(19–23) cmH_2_O	(18–21) cmH_2_O	
CVP	14.6 ± 1.6	12.2 ± 1.1	11.7 ± 1.3	0.016^*^
	(13–15) cmH_2_O	(11–14) cmH_2_O	(10–12) cmH_2_O	
Urine output per h.	1.2 ± 0.1	1.8 ± 0.2	1.9 ± 0.3	0.024^*^
	1–1.5 ml/kg/hr.	1.5–2 ml/kg/hr.	1.5–2.5 ml/kg/hr.	

*APP, Abdominal Perfusion Pressure; IVP, Intravesical pressure; PIP, Peak Inspiratory Pressure; CVP, Central Venous Pressure; P-values for immediate after complete closure vs. 24 h post-operative*;

* *Statistically significant*.

We used multivariate analysis to study the effect of multiple variables, such as age at operation, body weight, gestational age, size of the defect, IAP, PIP, CVP, and urine output per hour immediately after complete closure in correlation to each other and APP, and we found IAP, PIP, CVP, and urine are significant variables affecting APP. Any increase in these pressure parameters is associated with a decrease in APP. Also, in correlative studies of IAP values, APP was measured during operative trials of primary closure and was also assessed 24 and 48 h postoperatively and was observed to be significant statistically. This explains that intraoperative IAP measurement is a reliable parameter for the assessment of postoperative IAP that aids in the prevention of postoperative IAH, whereas PIP and CVP assessed during the trial of primary closure were correlated significantly with the IAP evaluated at the same time. Accordingly, PIP and CVP can be used as detectors as well as reliable predictors of IAH.

In regards to multivariate analysis of data collected on more than one variable, such as age at operation, body weight, gestational age, size of the defect, IAP, APP, PIP, and CVP immediate after complete closure in correlation with each other and mortality, we found a significant correlation between IAP, APP, PIP, CVP, and morality, and there was no significance with the rest of variables (Table 4).

**Table 4:** Multivariate analysis to study the effect of multiple variables in correlation to each other and mortality.

**Mortality**	**95.0% C.I. for odd**		***P*-value**
	**Lower**	**Upper**	
Age	0.986	1.051	0.265
Body weight	0.922	1.014	0.166
Gestational age	0.974	1.022	0.249
Size of defect	0.903	1.049	0.162
IAP immediate after complete closure	1.781	8.123	0.017^*^
APP immediate after complete closure	1.878	6.010	0.011^*^
PIP immediate after complete closure	9.013	14.353	0.018^*^
CVP immediate after complete closure	7.095	15.537	0.013^*^

*IAP, Intra-abdominal pressure; APP, Abdominal Perfusion Pressure; PIP, Peak Inspiratory Pressure; CVP, Central Venous Pressure*;

* *Statistically significant*.

We applied an umbilical cord flap in all cases. We had three categories of patients:

1. Prolene purse string was applied with daily tightening till complete closure in seven cases; none of them developed a ventral hernia (Figure 1).
2. Secondary suturing after shrinkage of the umbilical cord tissue due to loss of its water occurred in four cases. Taking the patient back to the OR after 7–10 days, remnants of the flap were removed. There are few adhesions between the gut and the cord tissue. The gut is further reduced by gentle pressure on monitoring the same previous parameters again. Mobilization of both skin and fascia was done, followed by closing with interrupted sutures.
3. Leaving skin creeping till complete closure of the defect occurred in nine cases. In seven cases, the abdominal defect was closed completely. Two patients who developed a ventral hernia required a secondary repair operation at 1.5 years old.

Postoperatively, IAH was not developed in any cases after our procedures. On the evaluation of cases 24 h after closure, no cases developed anuria, and we detected oliguria in two cases after 24 h; however, urine output showed a normal amount within the next 24 h. Mortality occurred in four (12.5%) cases out of all cases in the study (18), it was noticed that the highest percentage of mortality developed among patients with secondary suturing after shrinkage of the umbilical cord in three cases, and the fourth case was among the third category. Mortality was due to low birth weight, sepsis, and necrotizing enterocolitis. No infection was detected due to the umbilical cord flap.

## Discussion

Primary closure is not usually possible due to the viscero-abdominal disproportion, which may be a risk of ACS, and it is a big issue, especially in developing countries, where there are no available silos (19, 20). As regards silo placement, the reported complications include wound disruption, infection, adhesive intestinal obstruction, or compression by silo ring (21). Koltai used an umbilical cord flap to cover the herniated viscera like a silo, which leads to gradual sustained reduction with no adhesions or infection (22). Therefore, it has benefits of both a staged procedure in addition to the beneficial criteria of autogenic materials (23, 24).

A ventral hernia is proposed to occur using the umbilical cord flap as a proper covering tissue (17, 19). Our study showed only two patients developed ventral hernia that needed a secondary operation for repair at the age of 1.5 years; however, in Koltai's study, ventral hernia occurred in all cases (22).

The first step of the procedure is to reduce the bowel as much as possible. For the fear of ACS, we monitor the intraoperative urinary bladder pressure continuously. The limiting value of IVP in our study was 20 cmH_2_O, equivalent to 15 mmHg. On reaching this level during reduction of the viscera or testing of the approximation of the fascial edges, the umbilical cord is beneficial for coverage if preserved. According to Werbeck and Koltai, the limiting value was 20 mmHg (27 cmH_2_O), and they first used a full-thickness umbilical cord flap attached to its same site and then they split the cord to have more surface area without the removal of the vessels (23). In our study, we always split the cord and evacuated all its vessels to give a larger surface area, keeping it attached to its original base. Recently, in some of our cases, the umbilical cord was separated from its base using it as a “free graft” for better cosmoses of the ongoing umbilicus. There was no apparent difference about the cord side in contact with the viscera as no adhesions were noticed in both cases. We added a modification to the original method. Purse strings (Prolene Zero) were applied with daily tightening till complete closure in some cases, and none of them developed a ventral hernia.

In 1998, Bianchi used the umbilical cord flap for coverage as a part of a bedside reduction followed by suturing the umbilical cord remnant to the defect under local anesthesia without intubation soon after birth (25). As a modification, Sandler et al. covered the defect that was closed with the umbilical cord remnant by a clear plastic dressing. Also, they pulled the cord remnant across the defect and used adhesive tape to keep it in place, approximating the skin (26). Emami et al. considered that closure with a flap can be done initially or later on after gradual reduction of the viscera with a silo. Their study shows that closure using the flap was significantly more beneficial than the fascial closure; however, nearly half of flap patients underwent a previous silo trial as part of the definitive closure method. As a substitution in the case of the umbilical cord remnant shortage, simple skin reapproximation with adhesive tape supported with tincture of benzoin was also tried with success by one of their authors. An absorbent foam wrapped by a plastic dressing is used by another author (27).

Increased IAP due to a tight abdominal closure is associated with many drawbacks as the late recovery of bowel motility and also kidney affection. Lacey et al. illustrate in an animal study that IVP correlated with IAP, on rising above 15 mmHg, the renal blood flow impairs (20–40%), and cardiac output reduces by 20%; moreover, IAP > 25 mmHg impairs intestinal blood flow by 20–40%. IVP is considered a valuable parameter to perform primary or staged closure (28). Nakayama et al. illustrate a significant correlation between IVP and the inferior vena cava pressure as when it is still <20 mmHg, the complications could be prevented (29). Olesevich et al. report that full feeding return and shorter hospital stays were faster in cases whose primary closure was performed with IVP <20 mmHg (30). Chin et al. illustrate that closure with intraoperative IVP > 20 mmHg is associated with complications in the form of ascites, ventral hernia, impaired venous return of the lower extremities, and oliguria, and this complication is not encountered in neonates with an intraoperative IVP <20 mmHg (18). Lacey et al. avoided renal failure and oliguria in 48 neonates only when intraoperative IVP <20 mmHg (31). Santos Schmidt et al. and Rizzo et al. used a lower IVP threshold (20 cmH_2_O, equivalent to 15 mmHg) to choose the delayed primary closure or the staged approach (32, 33). Elsaied et al., use an IVP threshold of 20 cmH_2_O, but they did a routine postoperative assessment of IAP, and there was a statistically significant correlation between the values of IAP measured at primary abdominal closure and those measured 24 h postoperatively. They report that intraoperative IAP monitoring is a valuable predictor for the postoperative course, and consequently, it has a reliable role in the prevention of postoperative ACS. They observed that IAP values measured intraoperatively correlate significantly with both APP and PIP measured at the same time. Therefore, these parameters also can predict postoperative IAH/ACS (34).

Herein, we chose delayed primary closure vs. the staged approach using a lower IVP threshold (20 cmH_2_O, equivalent to 15 mmHg). We found higher values of APP in postoperative values than operative attempts of closure. The mean measurements of PIP, CVP in all measurements postoperatively showed lower values than operative attempts of closure, and urine output improved postoperatively in the following days. All these measurements show improvement in intestinal perfusion and a decrease in intra-abdominal pressure following closure using umbilical cord flap. We report less need for ventilator support, shorter time of total parenteral nutrition, and hospital stay. Also, the incidence of oliguria was still very low, reported in only two cases 24 h postoperatively; however, urine output retained normal values within the next 24 h.

In our study, mortality occurred in four (20%) cases, we observed that the highest percentage of mortality was among the patients with secondary suturing after shrinkage of the umbilical cord in three cases, and the fourth case was among the third category. Mortality was due to low birth weight, sepsis, and necrotizing enterocolitis. In low socioeconomic countries, the cases of death are high, reaching about half (35, 36). According to Watanabe et al., all patients who survived could be due to the advanced NICU care and nutritional support (37). In Ferdous et al., three of their patients (15%) died as they could not provide NICU support for all their patients in the form of available prolonged parenteral nutrition (38). According to Elsaied et al. and Shehata et al., the death rate was among patients for whom primary abdominal closure was done, which is low in comparison with the mortality percentage reported in patients repaired with silo (34, 39).

## Conclusion

The umbilical cord flap is feasible and should be considered as a cheap method for tension-free staged closure of gastroschisis. It is very effective to prevent the occurrence of ACS by limiting the PIP to 24 mmHg, IAP to 20 cmH_2_O (15 mmHg), APP > 50 mmHg, and CVP to 15 cmH_2_O. We need more patient numbers through a multicenter experience to verify the advantages of this method and to provide statistical power.

## Data Availability Statement

The original contributions presented in the study are included in the article/supplementary material, further inquiries can be directed to the corresponding author/s.

## Ethics Statement

The studies involving human participants were reviewed and approved by Faculty of Medicine, Tanta University, Egypt. Written informed consent to participate in this study was provided by the participants' legal guardian/next of kin. Written informed consent was obtained from the individual(s), and minor(s)' legal guardian/next of kin, for the publication of any potentially identifiable images or data included in this article.

## Author Contributions

MA contributed specifically in patients selection, partitioning, and operative technique. HS participated mainly in the study designing and the operative technique. KE participated in surgical technique and editing manuscript. HA operated cases and editing. MS participated in operative technique and statistical analysis. AE participated in surgical technique and follow up cases. All authors contributed equally in the writing in the manuscript and read and approved the manuscript for publications.

## Conflict of Interest

The authors declare that the research was conducted in the absence of any commercial or financial relationships that could be construed as a potential conflict of interest.

## Publisher's Note

All claims expressed in this article are solely those of the authors and do not necessarily represent those of their affiliated organizations, or those of the publisher, the editors and the reviewers. Any product that may be evaluated in this article, or claim that may be made by its manufacturer, is not guaranteed or endorsed by the publisher.
